# TRPC3 signalling contributes to the biogenesis of extracellular vesicles

**DOI:** 10.1002/jex2.132

**Published:** 2023-12-25

**Authors:** Elise H. Padbury, Štefan Bálint, Emanuela Carollo, David R. F. Carter, Esther B. E. Becker

**Affiliations:** ^1^ Nuffield Department of Clinical Neurosciences University of Oxford Oxford UK; ^2^ Kavli Institute for Nanoscience Discovery University of Oxford Oxford UK; ^3^ Kennedy Institute of Rheumatology University of Oxford Oxford UK; ^4^ Department of Biological and Medical Sciences Oxford Brookes University Oxford UK; ^5^ Evox Therapeutics Limited Oxford UK

**Keywords:** calcium, exosomes, Extracellular vesicles, histamine, ovarian cancer, TRPC3

## Abstract

Extracellular vesicles (EVs) contribute to a wide range of pathological processes including cancer progression, yet the molecular mechanisms underlying their biogenesis remain incompletely characterized. The development of tetraspanin‐based pHluorin reporters has enabled the real‐time analysis of EV release at the plasma membrane. Here, we employed CD81‐pHluorin to investigate mechanisms of EV release in ovarian cancer (OC) cells and report a novel role for the Ca^2+^‐permeable transient receptor potential (TRP) channel TRPC3 in EV‐mediated communication. We found that specific activation of TRPC3 increased Ca^2+^ signalling in SKOV3 cells and stimulated an immediate increase in EV release. Ca^2+^‐stimulants histamine and ionomycin likewise induced EV release, and imaging analysis revealed distinct stimulation‐dependent temporal and spatial release dynamics. Interestingly, inhibition of TRPC3 attenuated histamine‐stimulated Ca^2+^‐entry and EV release, indicating that TRPC3 is likely to act downstream of histamine signalling in EV biogenesis. Furthermore, we found that direct activation of TRPC3 as well as the application of EVs derived from TRPC3‐activated cells increased SKOV3 proliferation. Our data provides insights into the molecular mechanisms and dynamics underlying EV release in OC cells, proposing a key role for TRPC3 in EV biogenesis.

## 1 INTRODUCTION

Extracellular vesicles (EVs) are a heterogenous group of small membrane‐bound structures that are released into the extracellular environment by all studied cell types (Kowal et al., 2014; Record et al., 2011; Théry et al., 2002; Yáñez‐Mó et al., 2015). EVs and their accompanying biomolecular cargo including proteins, lipids and nucleic acids are taken up by neighbouring and distant cells, where they elicit significant pleiotropic effects on cell behaviour and are regarded as potent intercellular communicators in both normal physiological systems and disease (Hanayama, 2021; Yates et al., 2022a; Yates et al., 2022b). As mounting evidence indicates that EVs play a critical role in cancer progression (Dai et al., 2020; Gulei et al., 2018; Kahlert & Kalluri, 2013; Rak, 2010), understanding the factors that underly EV biogenesis and how these pathways can be manipulated holds significant therapeutic potential. This is particularly relevant to cancers with poor response rates to current therapeutics, such as ovarian cancer (OC), in which almost 45% of cases gain a chemoresistant phenotype following first‐line treatment (Galluzzi et al., 2012; Pokhriyal et al., 2019).

EVs are broadly classified into three main subpopulations based upon their mechanism of biogenesis and size: microvesicles (MVs), apoptotic bodies and exosomes. MVs and apoptotic bodies are shed by budding of the external plasma membrane (PM) from living and dying cells respectively, and can range in size between 100 and 1000 nm (Raposo & Stoorvogel, 2013; van Niel et al., 2018). Exosomes are comparatively smaller with a diameter between 40 and 150 nm and derive from the endocytic pathway as intraluminal vesicles (ILVs) in multivesicular bodies (MVBs) released upon MVB fusion with the PM (Harding et al., 1984; Klumperman & Raposo, 2014; van Niel et al., 2018). The biogenesis of MVBs and sorting of protein cargo into ILVs is mainly regulated by endosomal sorting complex required for transport (ESCRT) machineries and accessory proteins, including TSG101, ALIX, HRS and VPS4 (Henne et al., 2011). MVBs are then trafficked to the PM to release their constituents as exosomes through interactions with the actin and microtubule cytoskeleton, mediated by proteins of the Rab GTPase family (Blanc & Vidal, 2018; Sinha et al., 2016). Importantly, intracellular calcium (Ca^2+^) regulates ESCRT recruitment and MVB transport in endosomal release (Pang & Südhof, 2010; Scheffer et al., 2014), and several studies have shown that increases in intracellular Ca^2+^ induce EV release in cancer cell lines (Krämer‐Albers et al., 2007; Messenger et al., 2018; Savina et al., 2003). G protein‐coupled receptor (GPCR) signalling, which mediates Ca^2+^ entry, has also been implicated in the EV secretion pathway including activation of the histamine H_1_ receptor (H1HR) (Verweij et al., 2018). Furthermore, EVs released by cancer cells with disrupted Ca^2+^ signalling show enhanced metastatic capacity compared to EVs secreted under normal conditions (Lee et al., 2020; Messenger et al., 2018), suggesting that EVs may act as Ca^2+^‐driven downstream effectors in cancer progression. However, the molecular mechanisms that facilitate GPCR and Ca^2+^‐induced EV release remain incompletely understood.

The Ca^2+^ channel TRPC3 of the canonical transient receptor potential (TRPC) family is an essential regulator of intracellular Ca^2+^ homeostasis activated downstream of GPCR and receptor tyrosine kinase (RTK) stimulation at the PM (Kiselyov & Patterson, 2009; Lichtenegger & Groschner, 2014; Montell, 2005). Dysregulation of TRPC3 channel activity is associated with a broad spectrum of diseases (Nilius et al., 2007) including malignancy. TRPC3 is upregulated in numerous cancers and has been proposed to promote tumorigenesis by mediating sustained elevated Ca^2+^ signalling (Chang et al., 2018; Lin et al., 2021; Shapovalov et al., 2016; Wang et al., 2019; Yang et al., 2009). In OC, TRPC3 channels play a critical role in the proliferation and cell‐cycle progression of tumour cells in vitro and in vivo (Li et al., 2020; Tao et al., 2013; Yang et al., 2009). Interestingly, our previous work identified the dysregulation of several transcripts implicated in the biogenesis and secretion of EVs including *Unc13c, Smpd1, Snap23*, and *Tsg101* in the ataxic Moonwalker mouse model harbouring a gain‐of‐function mutation in TRPC3 (Dulneva et al., 2015).

Here, we report a novel role for TRPC3 in EV biogenesis in OC cells. Activation of TRPC3 increased Ca^2+^ signalling and EV release in SKOV3 cells. Using an optical reporter system in which the EV‐associated tetraspanin CD81 is fused with a pH‐sensitive GFP derivative, pHluorin (CD81‐pHluorin) (Verweij et al., 2018) to directly visualize EV release, we demonstrate distinct characteristics of EV release in SKOV3 cells following treatment with ionomycin, histamine and upon pharmacological activation of TRPC3. Notably, we find that Ca^2+^‐induced EV biogenesis in OC cells downstream of histamine is mediated by TRPC3. Finally, we show that EVs from TRPC3‐activated cells confer greater proliferative potential to recipient OC cells, suggesting a role for TRPC3‐mediated EV communication in the proliferation of OC cells. Together, our work provides insights into the dynamics of EV biogenesis and the molecular factors which facilitate EV release in cancer cells.

## 2 MATERIALS AND METHODS

### 2.1 Cell culture and materials

Human OC cell lines SKOV3, OVCAR3, OVCAR5 and IGROV1 were purchased from the National Cancer Institute, Frederick Cancer Division of Cancer Treatment and Diagnosis Tumour/Cell Line Repository (Bethesda, USA). The A2780 human OC cell line was kindly gifted by Professor Robert Brown, Imperial College, London, UK. All cell lines were cultured in RPMI 1640 medium (Lonza) supplemented with 10% (v/v) heat‐inactivated foetal bovine serum (FBS) (Gibco™) and maintained in humidified incubators at 37°C and 5% CO_2_. EV‐depletion of FBS was carried out by high‐speed ultracentrifugation in a SW32Ti swing‐bucket rotor (Beckman Coulter) at 120,000 *g* for 16 h at 4°C.

The selective TRPC3 agonist GSK1702934A (Xu et al., 2013) (Tocris) and TRPC3 antagonist PYR3 (Kiyonaka et al., 2009) (Sigma‐Aldrich®) were solubilized in DMSO, and serial‐diluted in the appropriate medium to concentrations of 0.1‐10 μM at the time of cell stimulation. An equivalent concentration of DMSO was applied to control cells. Histamine dihydrochloride (Sigma‐Aldrich®) and ionomycin calcium salt (Sigma‐Aldrich®) were solubilized in complete medium or Tyrode's solution, pH 7.4 (2 mM CaCl2, 2.5 mM KCI, 119 mM NaCl, 2 mM MgCl2, 30 mM glucose, 25 mM HEPES) to a concentration of 100 μM for histamine, and 1.25 μM ionomycin for all experiments.

Antibodies used for NFAT assays were rabbit polyclonal anti‐GFP (1:1,000, Novus Biologicals, NB600‐308) and secondary anti‐rabbit IgG (H+L) Alexa Fluor 594 (1:1,000, Thermofisher, A‐11012). For immunoblotting of TRPC3 in OC cell lines, rabbit anti‐TRPC3 (1:200, Alomone labs, ACC‐016) and mouse anti‐β‐actin (1:10,000, Proteintech, 66009‐1) were probed with horseradish peroxidase (HRP)‐conjugated anti‐mouse or anti‐rabbit secondary antibodies (1:10,000, GE Healthcare). Additional materials are described in Supplemental Methods and Table S1.

### 2.2 EV isolation

For all EV extractions, SKOV3 cells were cultured in a complete medium until ∼70% confluent, at which point cells were washed twice in PBS and switched to serum‐free media (SFM). Cells were conditioned in SFM for 48 h for EV characterization and 4 h for assessments of TRPC3‐mediated EV communication. Conditioned media was harvested and centrifuged at 300 *g* for 5 min at RT, followed by 16,500 *g* for 20 min at 4°C. The resulting supernatant was concentrated to 500 μL in Vivaspin 20 100 kDa concentrators (GE Healthcare) at 3000 *g* in a swing bucket rotor. EVs were extracted from the concentrate by size exclusion chromatography (SEC) in pre‐prepared columns (Biorad) with a 10 mL sepharose bed (Sepharose CL‐28, GE Healthcare). EV concentrate was loaded onto the column and eluted in sterile PBS, and 20 fractions of 500 μL flow‐through were collected. Fractions 5–10 were enriched for EVs as determined by Nanoparticle tracking analysis (NTA). These fractions were pooled and further concentrated to the desired volume for experiments in Vivaspin 2 5 kDa concentrators (GE Healthcare) at 3000 *g* in a swing bucket rotor. Methods for further characterization of EVs are described in Supplemental Methods.

### 2.3 Nanoparticle tracking analysis

EV concentration and size distribution were determined by NTA using a ZetaView PMX 110 (Particle Matrix) with NTA software v3.0. Data was acquired at RT with the following settings: sensitivity 80, frame rate 30 frames per second (fps), shutter speed 100, minimum brightness 25, maximum pixel size 1000 and minimum pixel size 5.

### 2.4 MACSPlex exosome assay

The MACSPlex Exosome Kit human (Miltenyi Biotec) is a multiplex bead‐based flow cytometric assay which enables semi‐quantitative detection of 37 EV surface markers. To characterize the molecular identity of EVs released upon TRPC3 activation, EVs were extracted from SKOV3 cells treated with 0.3 μM GSK1702934A or DMSO equivalent after 4 h conditioning. Following quantification by NTA, 1 × 10^9^ EVs from both treatment groups were diluted in MACSPlex buffer to a final volume of 120 μL and 15 μL of the MACSPlex Exosome Capture beads, and 15 μL of MACSPlex Exosome Detection Reagent cocktail, was added. Samples were then processed according to the overnight protocol for 1.5 mL reagent tubes published by the manufacturer. Analysis was performed with a CytoFLEX S flow cytometer (Beckman Coulter) and CytExpert v2.1 software, with 10,000 recorded events per sample. The mean fluorescence values were adjusted to the PBS control and normalized to CD63, CD81 and CD9 mean signal intensity.

### 2.5 EV treatment of cells

EVs were extracted as detailed from SKOV3 cells conditioned for 4 h in plain SFM (untreated control), or SFM supplemented with 3 μM PYR3, 0.3 μM GSK1702934A, or DMSO control (0.03%). SKOV3 cells were counted at the time of extraction and EVs were counted by NTA. Approximately 50 EVs per viable cell were counted at the time of EV extraction, therefore recipient cells were dosed with 50 particles per seeded cell at a 1x concentration, as well as 10× EVs/cell for cell growth analysis. An equivalent volume of PBS was applied to the ‘no EV’ control for each concentration. All recipient cells were seeded in complete medium, then washed twice in PBS and switched to EV‐depleted medium just prior to EV treatment to reduce contamination with FBS‐EVs.

### 2.6 Cell counts

To assess SKOV3 cell growth, cells were manually counted using a haemocytometer. In all experiments SKOV3 cells were seeded into 12‐well plates in complete medium at a density of 0.2 × 10^5^ cells. At time 0, cells were washed twice in PBS and switched to plain complete media (untreated control), or complete media supplemented with 0.3 μM GSK1702934A, 3 μM PYR3 or DMSO (0.03%). EV treatments were applied as detailed above. Cells were incubated in the respective treatment for 24, 48 or 72 h. At each timepoint cells were dissociated and pelleted, resuspended in 1 mL of complete media, and mixed at a 1:2 ratio with Trypan Blue (Biorad) to distinguish live from dead cells. A minimum of three wells were counted for every biological replicate in all experiments.

### 2.7 NFAT assays

NFAT assays were conducted as previously described (Hanson et al., 2015). SKOV3 cells seeded on coverslips were transfected with GFP‐tagged NFAT constructs (Addgene, 11107) using FuGENE HD transfection reagent (Promega) at a 3:1 FuGENE to DNA ratio, as indicated in the manufacturer's instructions. 24 h post‐transfection, cells were treated with 1.25 μM ionomycin, 0.1/0.3/1/10 μM GSK1702934A, 100 μM histamine, or a combination of histamine and 3 μM PYR3, alongside untreated and DMSO controls. In the combined treatment, cells were pre‐treated with PYR3 for 30 min prior to histamine stimulation. After 2 h incubation with the respective treatment, cells were fixed in 4% paraformaldehyde, washed in PBS and permeabilized with 0.4% Triton X‐100 (Sigma Aldrich®). Coverslips were then incubated with blocking solution (10% w/v skimmed milk powder and 1% v/v goat serum (Sigma Aldrich®) in Tris‐buffered saline with 0.2% Tween (Sigma Aldrich®) (TBST)) for 1 h at RT, followed by incubation overnight at 4°C in anti‐GFP primary antibody, and then 90 min at RT with secondary antibody. Coverslips were mounted onto glass slides with Prolong Gold Antifade 4′,6‐diamidin‐2‐fenilindolo (DAPI)‐containing mounting media (Thermofisher). Slides were imaged on a Zeiss AxioImager 2 upright microscope with the 20× objective, with five images taken per coverslip, three biological replicates per condition, and three technical replicates per biological replicate. ≥100 cells per technical replicate were analysed using FIJI software (v2.3.0) (Schindelin et al., 2012) to calculate the mean nuclear intensity/mean cytoplasmic intensity (N/C) ratio for each cell as described (Kelley & Paschal, 2019).

### 2.8 Immunoblotting

For cellular protein extraction, cells were washed in PBS and lysed in 1× radioimmunoprecipitation assay (RIPA) buffer (Sigma‐Aldrich®) in PBS supplemented with Halt™ protease inhibitor cocktail (1:1,000, Thermofisher). Lysates were incubated on ice for 10 min before pelleting by centrifugation at 14,000 *g* for 20 min at 4°C. Protein concentration was determined by Pierce BCA Protein Assay Kit (Thermofisher) according to the manufacturer's protocol.

Western blots were run using the Invitrogen NuPAGE system. For all samples, 50ug total protein was prepared in 4x LDS NuPAGE sample buffer (Invitrogen) supplemented with 0.4 mM dithiothreitol (DTT). Samples were boiled for 5 min at 95°C and loaded onto 4–12% polyacrylamide Bis‐Tris gels (Thermofisher) alongside 5 μL ladder (Biorad) and run at 125 V for 90 min in MES SDS buffer (Thermofisher). Proteins were transferred to a nitrocellulose membrane (0.45 μm pore, Amersham) by wet transfer for 2 h at 4°C, run at 150 V in 1x NuPAGE transfer buffer (Thermofisher) containing 15% methanol. Membranes were checked for protein transfer with Ponceau staining (Sigma‐Aldrich®), cut as required and blocked for 1 h at RT with 5% w/v skimmed milk powder in TBST (0.1% Tween). A higher concentration of 0.5% Tween was used for all blocking, antibody dilutions and washes for the detection of TRPC3. Membranes were incubated overnight at 4°C with primary antibodies diluted in blocking buffer, then washed three times for 5 min in TBST before incubating with secondary antibodies in blocking buffer for 1 h at RT. Washing steps were repeated, and membranes were incubated for 5 min with ECL Prime (Millipore Sigma) and imaged by ChemiDoc MP Bio‐Rad imaging system. Immunoblotting methods for EV markers are described in Supplemental Methods.

### 2.9 TIRF microscopy

The CD81‐pHluorin plasmid was kindly provided by Professor Aled Clayton, Cardiff University. SKOV3 cells were seeded in 8‐well glass bottom μ‐slides (Thistle Scientific) and transfected with CD81‐pHluorin using FuGENE HD transfection reagent (Promega) at a 3:1 FuGENE to DNA ratio, as indicated in the manufacturer's instructions. 24 h post‐transfection and just prior to imaging, cells were washed in Tyrode's solution, pH 5.5 (2 mM CaCl2, 2.5 mM KCI, 119 mM NaCl, 2 mM MgCl2, 30 mM glucose, 25 mM MES) to remove structures such as focal adhesions or exocytosed EVs from the coverslip. Cells were then imaged in Tyrode's solution, pH 7.4 in a 37°C humidified imaging chamber. For treatment conditions, Tyrode's solution, pH 7.4 was supplemented with 100 μM histamine, 1.25 μM ionomycin, 3 μM PYR3, 0.1/0.3/1 μM GSK1702934A, or DMSO control. For the analysis of histamine‐induced EV release in TRPC3 inhibited cells, cells were pre‐treated with PYR3 or DMSO for 30 min prior to histamine stimulation. For all treatment conditions, CD81‐pHluorin events were quantified in 5‐min acquisitions immediately following stimulation at a rate of 0.40 frames per second (fps). Cells expressing moderate levels of CD81‐pHluorin were selected for imaging, with 1 cell in the field of view per acquisition. A minimum of 10 cells were imaged per treatment condition. Live cell TIRF imaging was performed with an Olympus IX83 inverted microscope equipped with a 150x/1.45 oil‐immersion objective at 37°C. Fluorescent emission was collected by the same objective onto an electron‐multiplying charge‐coupled device camera (Evolve Delta, Photometrics).

### 2.10 Image analysis

All timeseries were analysed in FIJI (v2.3.0) (Schindelin et al., 2012) through a custom protocol. Initially, single channel image stacks were loaded into FIJI and formatted as 8‐bit for use with the StackDifference Plugin (Rietdorf & Seitz, 2008). The StackDifference Plugin was used to generate a sequence of difference images—each frame is the resultant subtraction of the next frame minus the previous frame. The gap between frames was set at 1. This method therefore selects for ‘new’ localized increases in fluorescence in every individual frame of the acquisition. The threshold was then adjusted to select for fluorescent events within the difference images stack, and the inbuilt ‘Analyze Particles’ function was employed to select and analyse fluorescent events above the set threshold. The fluorescence profile over‐time of every event highlighted by the Analyze Particles function was assessed with the Time Series Analyzer V3 Plugin (Balaji, 2014). Events with minimal change in fluorescence, an irregular fluorescence profile and/or lateral movement of the fluorescence signal were excluded from the analysis. For graphs demonstrating quantity of events, each data point represents the number of events in one cell. For descriptive data graphs, each data point represents a single event. Mean fluorescence values were taken directly from the ‘Analyze particles’ data. Width values were generated by manually measuring the diameter of fluorescent events at peak fluorescence in FIJI. The duration, localization and synchronization of CD81‐pHluorin events were assessed by eye.

### 2.11 Statistical analysis

All statistical analysis was performed in GraphPad Prism v6 software. Within the software, all data sets were assessed for normality using the Shapiro‐Wilk test, and the recommended parametric or non‐parametric alternative with multiple comparison test was conducted. All statistical tests are outlined in the figure legends. *p*‐Values in figures are shown as *p* < 0.05 *, *p* < 0.01 **, *p* < 0.001 *** and *p* < 0.0001 ****.

## 3 RESULTS

### 3.1 Real‐time visualization of EV release identifies distinct calcium‐ and histamine‐induced events in SKOV3 cells

**FIGURE 1:**
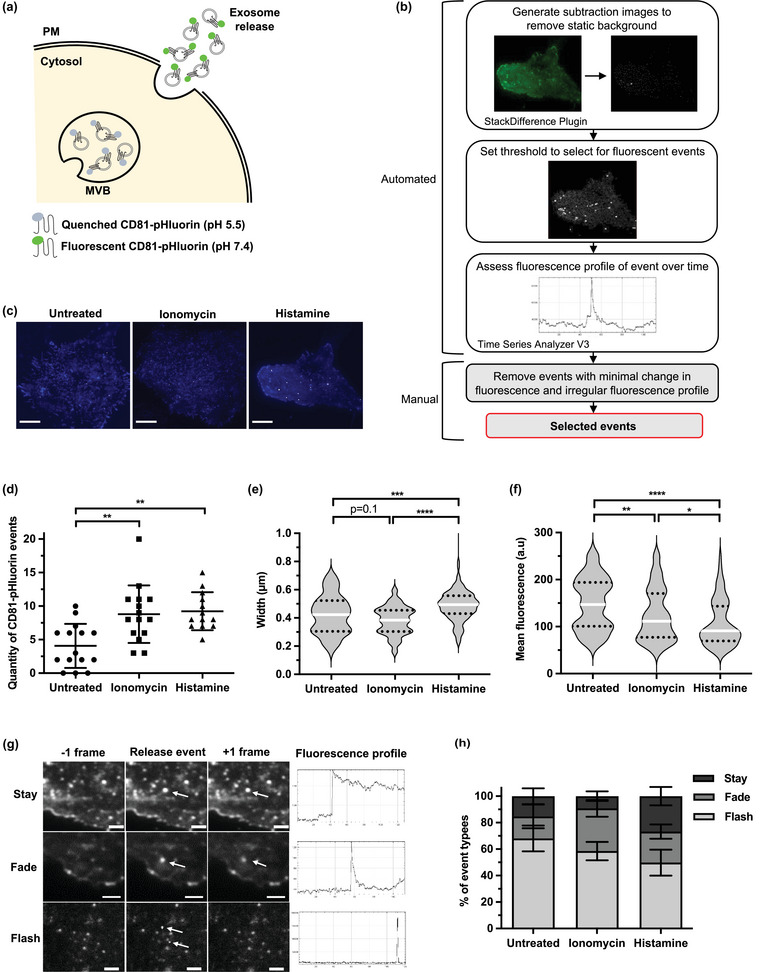
Histamine and ionomycin stimulate the release of CD81‐positive EVs with distinct characteristics and release dynamics in SKOV3 cells. (a) Schematic depicting the pHluorin reporter system: Within the acidic milieu of the multivesicular body (MVB), CD81‐pHluorin is quenched and thus non‐fluorescent. Upon fusion with the plasma membrane, MVB pH is neutralized and CD81‐pHluorin emits a bright, fluorescent signal. (b) Workflow for analysing fluorescent pHluorin events detected by TIRF microscopy using ImageJ. (c) Projection of total CD81‐pHluorin events in untreated cells (left) and cells treated with 1.25 μM ionomycin (middle) or 100 μM histamine (right) over a 5‐min time course. Pseudocoloured. Scale bars 10 μm. (d) Quantity of CD81‐pHluorin events captured in untreated, ionomycin and histamine treated cells represented in (C) over a 5‐min time course (mean ± SD; one‐way ANOVA followed by Tukey's multiple comparisons test; **p ≤ 0.01). See Videos S1‐S3. (e‐f) The width (e) and mean fluorescence (f) of CD81‐pHluorin events in untreated, ionomycin and histamine treated cells represented in (c) (violin plots show median and upper and lower quartiles; Kruskal‐Wallis followed by Dunn's multiple comparisons test; *p ≤ 0.05, **p ≤ 0.01, ***p ≤ 0.001, ****p ≤ 0.0001). (g) Representative images demonstrating stay, fade and flash event types over three consecutive frames, and their corresponding fluorescence profile over the total 5‐min time course. Arrows indicate CD81‐pHluorin events. Scale bars 2 μm. (h) Percentage of stay, fade and flash event types demonstrated in (g) in untreated, ionomycin and histamine treated cells over a 5‐min time‐course (mean ± SEM; two‐way ANOVA followed by Tukey's multiple comparisons test). (d‐h) represent a total of *n* ≥ 13 cells imaged over three independent experiments.

Tetraspanin‐based pHluorin reporters permit real‐time visualization of EV release in single cells by total internal reflection fluorescence (TIRF) microscopy (Figure 1a) and have previously been employed to demonstrate histamine‐ and ionomycin‐inducible signalling pathways for EV release (Verweij et al., 2018). To evaluate inducible EV biogenesis pathways in OC cells and validate our imaging and analysis protocols for downstream assessments of TRPC3 in EV biogenesis, we first assessed if histamine and ionomycin stimulate EV release in SKOV3 cells and characterized their release dynamics. We selected CD81‐pHluorin as a reporter of EV release in SKOV3 cells as immunogold transmission electron microscopy (TEM) of SKOV3‐EVs detected an abundance of particles typical of EV morphology and size that labelled positive for CD81, which was confirmed by immunoblotting (Figure S1‐S3). Immunocytochemistry in SKOV3 cells confirmed that CD81 colocalizes with early and late endosomal markers RAB5 and RAB7 respectively, in addition to being expressed at the PM (Figure S4). As the fluorescence of the CD81‐pHluorin reporter is dependent on the pH switch upon MVB‐PM fusion, visualized events should largely represent the release of CD81+ exosomes. To quantify CD81+ EV release, an analysis workflow was developed in ImageJ selecting for ‘new’ localized increases in fluorescence above a set threshold in every individual frame of the acquisition (Figure 1b). The fluorescence profile of all selected events was manually assessed, and events with minimal change in fluorescence, an irregular fluorescence profile and/or lateral movement of the fluorescence signal were excluded from the analysis. CD81‐pHluorin events were quantified in SKOV3 cells stimulated with 100 μM histamine or 1.25 μM ionomycin in 5‐min acquisitions immediately following stimulation. Both ionomycin and histamine significantly increased the rate of CD81‐pHlourin events compared to untreated controls (Figure 1c,d and Videos S1‐S3). This is consistent with previous studies reporting an increase in CD63‐pHlourin events upon histamine stimulation of HeLa and HUVEC cells (Verweij et al., 2018), and ionomycin stimulation of the breast cancer cell line MDA‐MB‐231 (Messenger et al., 2018).

**FIGURE 2:**
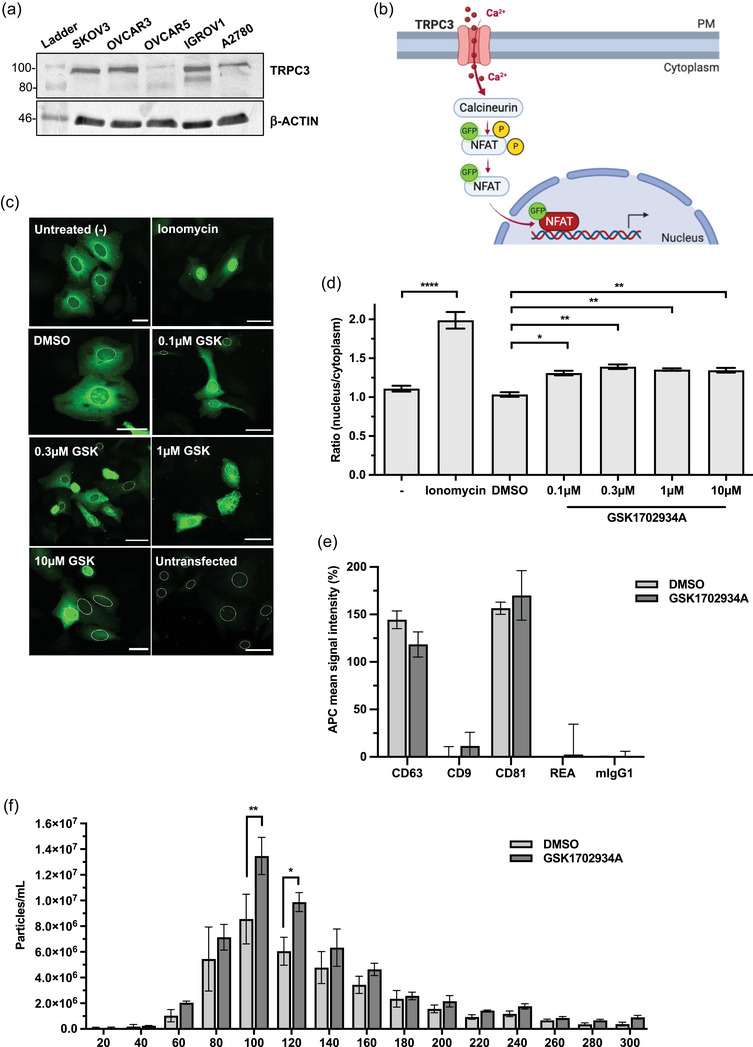
TRPC3 activation increases calcium signalling and EV release in SKOV3 cells. (a) Western blot of endogenous TRPC3 and β‐actin protein expression in five ovarian cancer cell lines: SKOV3, OVCAR3, OVCAR5, IGROV1 and A2780. Representative image of three independent experiments. (b) Schematic representation of NFAT translocation assay. Activation of TRPC3 stimulates a rise in intracellular calcium (Ca^2+^) which subsequently activates the phosphatase calcineurin, inducing dephosphorylation and nuclear translocation of NFAT which can be visualized by a GFP tag. (c) Representative images of NFAT translocation in SKOV3 cells expressing GFP‐tagged NFAT following treatment with 1.25 μM ionomycin or 0.1/0.3/1/10 μM GSK1702934A, alongside untreated (‐) and DMSO controls. Fixed cells were subjected to indirect immunofluorescence using an anti‐GFP antibody and the DNA dye DAPI. Nuclear regions are indicated. Scale bars 50 μm. (d) Nuclear/cytosol fluorescence intensity ratio of treated cells shown in (c) (mean ± SEM of ≥100 cells over three independent experiments; one‐way ANOVA followed by Tukey's multiple comparisons test; *p ≤ 0.05, **p ≤ 0.01, ****p ≤ 0.0001). (e) MACSPlex exosome assay data showing the mean signal intensities of CD63, CD9, CD81 and two isotype controls detected on EVs isolated from SKOV3 cells treated with 0.3 μM GSK1702934A or DMSO equivalent. APC fluorescence intensity adjusted to PBS control. Mean ± SEM of three independent isolations. (f) NTA analysis of EVs isolated from SKOV3 cells treated with 0.3 μM GSK1702934A or DMSO equivalent (mean ± SEM of three independent isolations; two‐way ANOVA followed by Tukey's multiple comparisons test; *p ≤ 0.05, **p ≤ 0.01).

To further characterize histamine‐ and ionomycin‐induced EV release and gain more information on their release dynamics, all CD81‐pHlourin events were assessed by size, mean fluorescence and duration. Histamine‐induced events were significantly larger and dimmer than both ionomycin‐stimulated and basal events (Figure 1e,f). Ionomycin‐induced events were also significantly dimmer than basal events (Figure 1f). We also observed differences in the duration of fluorescence events. All fluorescence events were characterized by a sudden increase in localized fluorescence, but the decay kinetics differed between event types, which we classified as stay, fade, and flash, respectively (Figure 1g). Typically, stay events did not decrease in fluorescence over the duration of the acquisition (5 min), fade events decayed exponentially over time, and flash events returned to background fluorescence within a single frame. The proportions of observed event types differed under histamine and ionomycin stimulation (Figure 2h). Basal EV release consisted of largely flash event types (68%) with a smaller, and equal proportion of fade and stay events (∼16%). Fade event types were most evident in ionomycin‐stimulated cells (32%), with only 10% of ionomycin‐induced EV release consistent with stay event types. Conversely, histamine‐stimulated cells had the largest proportion of stay events (27%) and less than 50% flash event types. Taken together, this data demonstrates that histamine and ionomycin induce CD81‐pHlourin events with distinct characteristics and dynamics, possibly representing independent EV biogenesis pathways or the release of distinct EV sub‐populations.

### 3.2 Pharmacological activation of TRPC3 increases calcium signalling and EV release in SKOV3 cells

A gain‐of‐function mutation in TRPC3 results in increased Ca^2+^ signalling and expression changes in genes implicated in EV biogenesis and secretion in the Moonwalker mouse model (Dulneva et al., 2015). We therefore set out to investigate whether TRPC3 might play a role in EV biogenesis. We first measured the endogenous expression of TRPC3 across a panel of epithelial OC cell lines; SKOV3, OVCAR3, OVCAR5, IGROV1, and A2780. Endogenous TRPC3 protein expression was detected in all OC lines except OVCAR5 by Western blot (Figure 2a, Figure S3). As the SKOV3 cell line has previously been used to investigate the role of TRPC3 in OC as well as in studies of EV release (Escrevente et al., 2011; Kobayashi et al., 2014; Yang et al., 2009; Zeng et al., 2013), we selected this cell line for further study.

The selective TRPC3 agonist GSK1702934A was employed to activate endogenous TRPC3 in SKOV3 cells. To assess downstream changes in Ca^2+^ signalling and as a readout for TRPC3 activity (Fogel et al., 2015; Hanson et al., 2015), cells were transfected with a GFP‐tagged version of the Ca^2+^‐sensitive transcription factor NFAT. Under normal conditions inactive, phosphorylated NFAT resides in the cytoplasm, but upon Ca^2+^ influx, NFAT is dephosphorylated by Calcineurin and translocates to the nucleus (Hogan et al., 2003) (Figure 2b). SKOV3 cells expressing GFP‐tagged NFAT were treated for 2 h with 1.25 μM ionomycin as a positive control or increasing concentrations of GSK1702934A, alongside untreated (‐) and DMSO controls. Ionomycin stimulated the most significant increase in NFAT nuclear translocation, and all concentrations of TRPC3 activator significantly increased the nuclear/cytosol (N/C) fluorescence intensity ratio compared to the DMSO control (Figure 2c,d). A modest dose‐dependent response was apparent up to concentrations of 0.3 μM, whereafter the effect on Ca^2+^ influx appeared to be saturated. GSK1702934A has been shown to selectively activate TRPC3 with an EC_50_ value of 0.08 μM (Xu et al., 2013), therefore 0.3 μM likely represents maximum channel activation in SKOV3 cells.

As increases in intracellular Ca^2+^ can induce EV release (Krämer‐Albers et al., 2007; Messenger et al., 2018; Savina et al., 2003), we next assessed the effect of activating TRPC3 on EV release from SKOV3 cells. Western blotting of SKOV3‐EVs extracted by size exclusion chromatography (SEC) confirmed the presence of EV markers CD81, CD9, TSG101 and ALIX, whilst the mitochondrial marker cytochrome C and Golgi marker GM130 were absent (Figure S1), suggesting that EV preparations were clear of cellular contamination. To characterize EVs released upon TRPC3 activation, EVs were extracted from SKOV3 cells treated with 0.3 μM GSK1702934A or DMSO equivalent after 4 h conditioning. MACSPlex analysis detected high expression of CD63 and CD81 and minimal CD9 in both treatment groups (Figure 2e), suggesting that TRPC3 activation does not alter the molecular identity of released EVs. However, quantification of EVs by nanoparticle tracking analysis (NTA) demonstrated that TRPC3 activator‐treated cells released significantly more EVs than DMSO‐treated cells (Figure 2f), indicating that TRPC3 activation increases EV release in SKOV3 cells. Together, these data indicate that TRPC3 activation affects Ca^2+^ signalling and downstream EV release in SKOV3 cells.

### 3.3 TRPC3 activation stimulates the release of distinct EVs in SKOV3 cells

**FIGURE 3:**
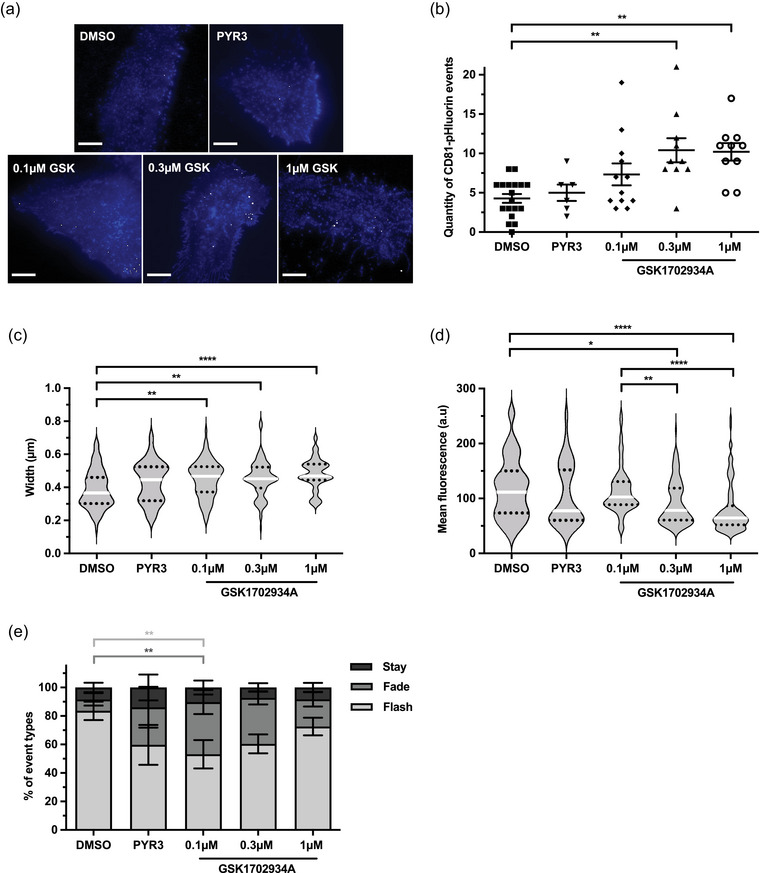
TRPC3 activation stimulates distinct EV release. (a) Projection of total CD81‐pHlourin events in DMSO control and treated cells over a 5‐min time course. Top row, left to right: DMSO control and 1 μM PYR3. Bottom row, left to right: 0.1, 0.3 and 1 μM TRPC3 activator GSK1702934A (GSK). Pseudocoloured. Scale bars 10 μm. (b) Quantity of CD81‐pHluorin events captured in DMSO control and treated cells represented in (a) over a 5‐min time course (mean ± SEM; one‐way ANOVA followed by Tukey's multiple comparisons test; **p≤0.01). See Video S4. (c‐d) The width (c) and mean fluorescence (d) of CD81‐pHluorin events in DMSO control cells, and cells treated with 1 μM PYR3 or 0.1, 0.3, 1 μM GSK1702934A (violin plots show median and upper and lower quartiles; Kruskal‐Wallis followed by Dunn's multiple comparisons test; *p ≤ 0.05, **p ≤ 0.01, ****p ≤ 0.0001). (e) Percentage of stay, fade and flash event types in DMSO control and treated cells in (a) over a 5‐min time‐course (mean ± SEM; two‐way ANOVA followed by Tukey's multiple comparisons test; **p ≤ 0.01). (b‐e) represent a total of n≥6 cells imaged over three independent experiments.

To further characterize the effect of TRPC3 stimulation on EV biogenesis, we quantified CD81‐pHluorin events in SKOV3 cells treated with increasing concentrations of GSK1702934A, the TRPC3 inhibitor PYR3, or DMSO control. CD81‐pHluorin events were quantified in 5‐min acquisitions immediately following stimulation. PYR3‐treated cells showed no difference in EV release compared to the DMSO control (Figure 3a,b). In contrast, the quantity of CD81‐pHluorin events significantly increased in a dose‐dependent manner following TRPC3 activation (Video S4), consistent with a role for TRPC3 in EV release in SKOV3 cells.

To understand how modulating TRPC3 activity may affect the characteristics and dynamics of CD81‐pHluorin events, all events were assessed by size, mean fluorescence, and duration. PYR3 demonstrated no effect on the characteristics of CD81 events (Figure 3c‐e). However, at increasing concentrations of GSK1702934A and thus increasing rates of TRPC3‐mediated EV release, CD81‐pHluorin events were significantly larger and dimmer than events observed in DMSO control cells in a dose‐dependent manner (Figure 3c,d). Interestingly, these were the same characteristics that we observed in histamine‐treated cells (Figure 1e,f). Upon classifying CD81‐pHluorin events by their duration into stay, fade, or flash event types, we observed differences in the proportions of observed event types under TRPC3 modulation (Figure 3e). DMSO‐induced EV release mainly consisted of flash event types (63% and 84% respectively) with a smaller proportion of fade events. Stay event types were consistent across all treatment groups varying between 7–14% of captured events. Notably, TRPC3 activation with 0.1 μM GSK1702934A induced significantly more fade event types and fewer flash events compared to the DMSO control. Collectively these data demonstrate distinct EV characteristics and release dynamics in OC cells upon TRPC3 activation.

### 3.4 Induced EV release in SKOV3 cells is localized and synchronized

**FIGURE 4:**
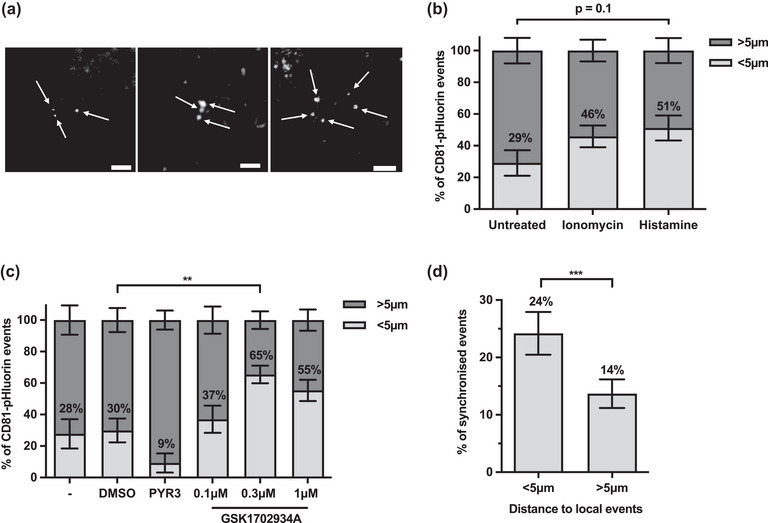
EV release in SKOV3 cells is localized and synchronized. (a) Representative images demonstrating instances of localized CD81‐pHluorin events in SKOV3 cells, indicated by white arrows. Scale bars 2 μm. (b‐c) Percentage of localized CD81‐pHluorin events (defined as events occurring within 5 μm (<5 μm) of another event in the same cell) in (b) untreated, ionomycin‐ and histamine‐treated cells and (c) untreated (‐), DMSO, PYR3 or 0.1, 0.3, 1 μM GSK1702934A‐treated cells (mean ± SEM; a total of *n* ≥ 6 cells imaged over three independent experiments; two‐way ANOVA followed by Tukey's multiple comparisons test; **p ≤ 0.01, ***p ≤ 0.001). (d) Percentage of synchronized events, which occur within 5 μm (<5 μm) or further away than 5 μm (>5 μm) of another CD81‐pHluorin event in the same cell (mean ± SEM of all imaged cells regardless of treatment condition, *n* = 101; Wilcoxon matched‐pairs signed rank test; ***p ≤ 0.001).

Single‐cell analysis of CD81‐pHluorin events allowed us to gain further insights into the subcellular localization of EV release. During the analysis of EV release events, we noted that CD81‐pHluorin events regularly occur within close proximity of one another—particularly in stimulated conditions (Figure 4a). The distance between events within the same cell was subsequently measured in all treatment conditions, and events were considered localized if they occurred within 5 μm of another event. In histamine‐ and ionomycin‐treated cells a higher proportion of CD81‐pHluorin events were localized compared to basal EV release (46% and 51% for ionomycin‐ and histamine‐induced events respectively, vs. 29% for basal events) (Figure 4b). Similarly, TRPC3 activation significantly increased the number of localized events in SKOV3 cells compared to DMSO‐induced EV release—peaking at 65% at 0.3 μM activator vs. 30% for DMSO. Interestingly, although TRPC3 inhibition with PYR3 showed no effect on the quantity or characteristics of EV release (Figure 3a‐e), localized release was reduced to 9% in PYR3‐treated cells.

We also noted that localized CD81‐pHluorin events often occurred simultaneously. To quantify this effect, we measured the distance between all events that appeared in the same frame and assessed the percentage of synchronized events that were localized or diffuse. Significantly more localized events appeared simultaneously compared to diffuse release ‐ 24% to 13% respectively (Figure 4d). This effect was not treatment‐dependent (data not shown). Overall, these results demonstrate that treatment with histamine, ionomycin and GSK1702934A induce localized and synchronized CD81‐pHluorin events in SKOV3 cells.

### 3.5 Histamine induces EV release through activation of the TRPC3 channel

**FIGURE 5:**
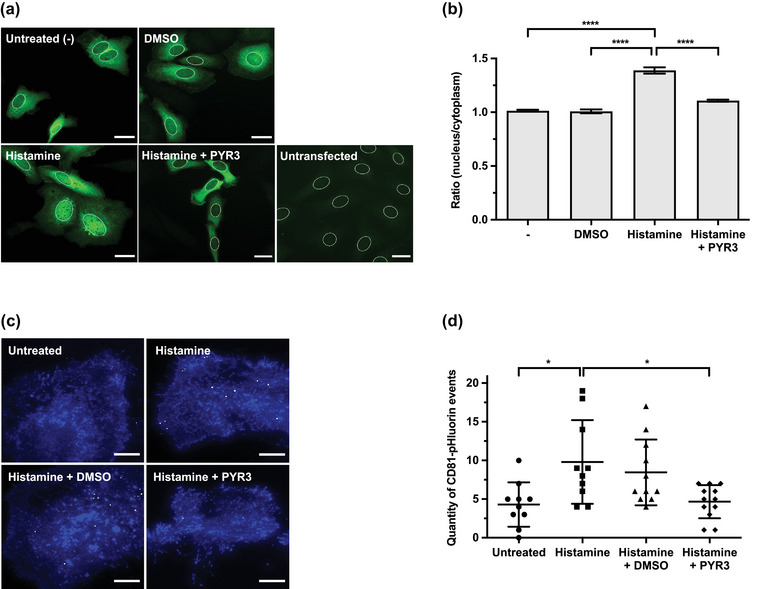
TRPC3 inhibition attenuates histamine‐stimulated calcium entry and EV release. (a) Representative images of NFAT translocation in SKOV3 cells expressing GFP‐tagged NFAT following treatment with 100 μM histamine or 100 μM histamine plus 3 μM PYR3, alongside untreated (‐) and DMSO controls. Fixed cells were subjected to indirect immunofluorescence using an anti‐GFP antibody and the DNA dye DAPI. Nuclear regions are indicated. Scale bars 50 μm. (b) Nuclear/cytosol fluorescence intensity ratio of treated cells shown in (a) (mean ± SEM of ≥100 cells over three independent experiments; one‐way ANOVA followed by Tukey's multiple comparisons test; ****p ≤ 0.0001). (c) Projection of total fusion events in untreated cells (top left) and cells treated with 100 μM histamine (top right), 100 μM histamine + DMSO (bottom left) or 100 μM histamine + 0.3 μM PYR3 (bottom right) over a 5‐min time course. Pseudocoloured. Scale bars 10 μm. (d) Quantity of CD81‐pHluorin events captured in untreated cells, and cells treated with histamine, histamine + DMSO and histamine + PYR3 represented in (C) over a 5‐min time course (mean ± SD; *n* ≥ 10 cells over two independent experiments; one‐way ANOVA followed by Tukey's multiple comparisons test; *p ≤ 0.05).

Histamine stimulation of HeLa cells induces EV release through GPCR signalling from H1HR (Verweij et al., 2018). Downstream of H1HR activation, phospholipase C (PLC) stimulates diacylglycerol (DAG) and inositol 1,4,5‐trisphosphate (IP_3_) production through the phosphatidylinositol signalling pathway (Hill et al., 1997; Panula et al., 2015). TRPC3 is activated in response to IP_3_‐mediated Ca^2+^ release from internal stores and DAG (Kiselyov & Patterson, 2009; Montell, 2005), and has been reported to function downstream of H_1_ and H_2_ receptors in some cell types to facilitate histamine‐induced Ca^2+^ entry (Hofmann et al., 1999; Kwan et al., 2009). As both histamine and TRPC3 activation stimulate EV release in OC cells, we next investigated whether histamine induces EV release through downstream activation of the TRPC3 channel. We first showed that H1HR is highly expressed at both the mRNA and protein level in SKOV3 cells (Figure S5), which is consistent with gene expression data from the Cell Miner database (GSE32474) showing that H1HR is the predominantly expressed histamine receptor in SKOV3 cells (Reinhold et al., 2012). To establish if histamine stimulation increases Ca^2+^ signalling in SKOV3 cells and if this effect is mediated by TRPC3, Ca^2+^ signalling was evaluated in SKOV3 cells stimulated with 100 μM histamine, and in SKOV3 cells pre‐incubated for 30 min with 3 μM TRPC3 inhibitor PYR3 prior to histamine stimulation. Histamine significantly increased the nuclear localization of GFP‐tagged NFAT compared to untreated and DMSO controls, indicating that histamine stimulation increases Ca^2+^ signalling in SKOV3 cells (Figure 5a,b). In histamine‐stimulated cells that were pre‐treated with TRPC3 inhibitor, the effect of histamine was significantly reduced to control levels. These findings suggest that TRPC3 mediates Ca^2+^ signalling downstream of histamine stimulation in SKOV3 cells.

Next, we quantified CD81‐pHluorin events in SKOV3 cells stimulated with 100 μM histamine, and SKOV3 cells pre‐incubated for 30 min with 3 μM TRPC3 inhibitor PYR3 or DMSO control prior to histamine stimulation. Pre‐incubating SKOV3 cells with DMSO did not inhibit histamine‐stimulated EV release. However, in histamine‐stimulated cells pre‐treated with PYR3, the effect of histamine was diminished, significantly reducing CD81‐pHluorin events to that of the untreated control (Figure 5c,d). Collectively, these results suggest that histamine and TRPC3 activation function in a shared, Ca^2+^‐dependent EV release pathway in which TRPC3 acts downstream of histamine activation.

### 3.6 TRPC3‐mediated EV release contributes to the proliferation of ovarian cancer cells

**FIGURE 6:**
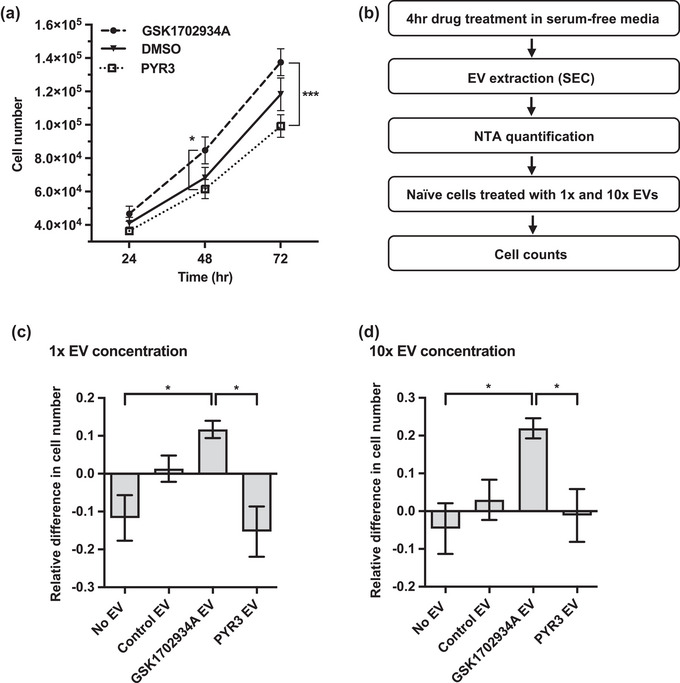
TRPC3 activation and TRPC3 activator‐induced EVs drive SKOV3 cell growth. (a) Growth curves representing total cell number at 24, 48 and 72 h incubation with TRPC3 activator GSK1702934A (0.3 μM), TRPC3 inhibitor PYR3 (3 μM) or DMSO control (mean ± SEM; *n* = 4; two‐way ANOVA followed by Tukey's multiple comparisons test; *p ≤ 0.05, ***p ≤ 0.001). (b) Experimental procedure to assess TRPC3‐mediated EV communication: SKOV3 cells were grown to ∼80% confluency in complete media, washed and treated with 0.3 μM TRPC3 activator, 3 μM TRPC3 inhibitor or DMSO equivalent in serum‐free media (SFM) for 4 h. EVs were then isolated from the conditioned media and quantified by NTA. 1× and 10× EV/cell concentrations were calculated from the untreated control and added to naïve cells in media supplemented with 10% EV‐depleted FBS. Equivalent volumes of PBS were added to cells as a no EV control. Cell counts with Trypan Blue were used to assess the effects of TRPC3‐mediated EV communication. (c‐d) The relative difference in total cell number at 48 h between all treatment groups and DMSO‐treated control EVs at 1× and 10× EV treatment concentrations (mean ± SEM; *n* = 3; one‐way ANOVA followed by Tukey's multiple comparisons test; *p ≤ 0.05).

Increased activity of TRPC3 is thought to contribute to the progression of OC through mediating sustained, elevated Ca^2+^ signalling (Yang et al., 2009), however roles for Ca^2+^‐dependent downstream effectors are poorly understood. Here, we showed that TRPC3 activation increased Ca^2+^ signalling in OC cells and induced the biogenesis of a characteristically unique EV population. Given that EVs released by cancer cells with disrupted Ca^2+^ signalling can exhibit enhanced metastatic capacity (Lee et al., 2020; Messenger et al., 2018), we hypothesized that EVs induced by TRPC3 activation may contribute to OC tumorigenesis through enhancing cell proliferation. We first assessed the effects of modulating TRPC3 activity on OC cell growth. SKOV3 cells were counted at 24, 48, and 72 h following treatment with 0.3 μM TRPC3 activator or 3 μM TRPC3 inhibitor. The number of TRPC3 activator‐treated cells was consistently higher than control‐treated cells and significantly increased in comparison to TRPC3 inhibitor‐treated cells at both 48 and 72 h (Figure 6a). This is in line with previous studies demonstrating that TRPC3 activation accelerates cell growth (Li et al., 2020; Tao et al., 2013; Yang et al., 2009). To determine if there is any effect of EVs released from TRPC3 activator‐treated cells on cell growth, EVs were extracted from all treatment groups after 4 h conditioning and quantified by NTA, before being added to naïve SKOV3 cells (Figure 6b). The number of EVs released per cell was calculated based on NTA quantification and recipient cells were treated with an equivalent number of EVs per seeded cell (1x) or at 10x concentration of EVs added per seeded cell. At 48 h, EVs released from TRPC3‐activated cells significantly increased recipient cell growth at both 1x and 10x dosage compared to the no EV control (Figure 6c,d, Figure S6). In contrast, EVs released from cells that were treated with PYR3 inhibitor consistently reduced recipient cell growth, although this effect was only significant in comparison to TRPC3 activator‐EVs. These results suggest that EVs released upon TRPC3 activation may possess the functional capacity to alter the growth rate of recipient SKOV3 cells.

## 4 DISCUSSION

With EVs emerging as critical cell‐cell communicators in cancer progression (Dai et al., 2020; Gulei et al., 2018; Kahlert & Kalluri, 2013; Rak, 2010), uncovering the molecular mechanisms and dynamics which underlie EV biogenesis holds significant therapeutic potential. GPCR activation and elevated intracellular Ca^2+^ have been shown to stimulate EV release in cancer cells and alter their metastatic capacity (Lee et al., 2020; Messenger et al., 2018; Verweij et al., 2018), but regulatory proteins that facilitate downstream EV biogenesis have not been identified. In this study, we show that activation of the Ca^2+^ channel TRPC3 increased Ca^2+^ signalling and EV release in OC cells, demonstrating a novel role for TRPC3 in EV biogenesis. Moreover, we have characterized distinct EV release dynamics following activation by TRPC3 activation as well as histamine and ionomycin. Interestingly, our findings suggest that histamine and TRPC3 activation stimulate a shared, Ca^2+^‐dependent EV release pathway. Finally, EVs induced by activation of TRPC3 conferred greater proliferative potential to recipient OC cells, collectively proposing a potential role for TRPC3‐mediated EV communication in OC.

Tetraspanin‐based pHluorin reporters have previously been employed to demonstrate histamine‐ and ionomycin‐inducible signalling pathways for EV release (Messenger et al., 2018; Verweij et al., 2018). Here, using CD81‐pHluorin, we show for the first time that histamine and ionomycin induce EV release in OC cells. Moreover, we find that histamine and ionomycin‐induced CD81‐pHluorin events differ in size, fluorescence intensity and duration. These stimulant‐dependent characteristics suggest that histamine and ionomycin may stimulate distinct biogenesis pathways in OC cells, or the release of different EV sub‐populations. Previous work on the classification of EV subtypes by the duration of the fluorescent signal (Verweij et al., 2018) suggests that observed fade event types might correspond to true MVB‐PM fusion for exosome release. These data also suggest that the short‐lived fluorescence corresponding to the observed flash event types might be associated with the release of soluble cargo or membrane deposition (Verweij et al., 2018). Stay event types might represent incomplete exosome exocytosis whereby the vesicle pore transiently opens and closes without fully collapsing into the PM, or fragments of the endosomal membrane are incorporated into the PM as ‘blebs’ or ‘patches’. However, absolute fusion duration times will vary between cell types, temperature conditions, medium viscosity etc., therefore any comparisons of relative fusion duration should ideally be determined for each cell line and individual experiment. Additional mechanisms may also contribute to the variable characteristics of induced CD81‐pHluorin events. For example, histamine and ionomycin may influence the loading of CD81 into EVs, therefore generating the dimmer CD81‐pHluorin events observed. Similarly, larger histamine‐induced events may contain less CD81‐positive ILVS, resulting in a more diffuse fluorescent signal. Moreover, the size of treatment‐induced EVs may translate to the observed differences in signal duration, with larger histamine‐induced EVs emitting more prolonged fluorescent fade and stay events than unstimulated EV release. Entrapment of EVs between cells and the coverslip and interactions with the extracellular matrix may also account for differences in fluorescent signal duration. In future experiments, TEM or correlative light and electron microscopy (CLEM) could be used to validate the inferred morphology and duration of pHluorin signals and improve our understanding of their biological significance.

In this study, we used CD81‐pHluorin to investigate a role for TRPC3 in EV biogenesis in OC. In SKOV3 cells, pharmacological activation of TRPC3 increased Ca^2+^ signalling and induced the release of 100–120 nm EVs, which were positive for EV markers CD63 and CD81. Analysis of CD81‐pHluorin events confirmed that TRPC3 activation increased the rate of EV release in SKOV3 cells. This is the first direct demonstration of TRPC3 involvement in EV biogenesis. Treatment with the TRPC3 inhibitor PYR3 did not reduce the quantity of CD81‐pHluorin events, likely due to the already low resting rate of CD81‐pHluorin events in SKOV3 cells. Interestingly, CD81‐pHluorin analysis also detected a size‐dependent increase in EV release, as TRPC3 activator‐induced EVs were significantly larger than control EVs in a dose‐dependent manner. This suggests that TRPC3 activation may stimulate Ca^2+^‐regulated molecules such as Rab11, which is known to generate Ca^2+^‐induced giant MVBs (Savina et al., 2005). Alternatively, TRPC3 activation may induce the release of a specific EV subpopulation as shown for the sphingomyelinase inhibitor GW4869, which specifically increased the release of larger EVs (100‐200 nm) (Mathieu et al., 2021; Menck et al., 2017).

In addition to altering the frequency and characteristics of CD81‐pHluorin events, stimulating SKOV3 cells induced sites of localized and synchronized EV release—which we have termed “EV hotspots”. Instances of regulated and localized EV release have previously been described (Mittelbrunn et al., 2011; Sung et al., 2020; van Niel et al., 2001) and may have important biological consequences in vivo. For example, at the immunological synapse between B and T cells, GPCRs CCR5 and CXCR4 required for T cell activation are recruited to sites of localized EV release (Contento et al., 2008; Mittelbrunn et al., 2011). We propose a similar mechanism for histamine‐ and TRPC3‐induced EV release, whereby EV hotspots may coincide with sites of histamine‐GPCRs or TRPC3 channels at the PM. Future experiments to map sites of localized EV release to subcellular structures and to assess co‐localization with PM‐associated proteins will provide deeper insights into EV hotspots in OC.

TRPC3 activity is regulated by GPCR stimulation of the phosphatidylinositol signalling pathway (Kiselyov & Patterson, 2009; Montell, 2005) and has been reported to function downstream of histamine H_1_ and H_2_ receptors to facilitate histamine‐induced Ca^2+^ entry (Hofmann et al., 1999; Kwan et al., 2009). Our results show that TRPC3 mediates histamine‐induced Ca^2+^ entry and EV release in SKOV3 cells. In addition, histamine‐ and TRPC3 activator‐induced event types were similar by TIRF microscopy (larger, and dimmer than control events). These findings suggest that histamine and TRPC3 activation stimulate a shared, Ca^2+^‐dependent pathway, mediated by H1HR‐TRPC3‐phosphatidylinositol signalling. Identification of this shared pathway is significant for understanding the underlying mechanisms for both EV biogenesis and Ca^2+^‐signalling in OC cells, although further investigation of the nature of the involved histamine GPCR is required. Our findings are contradictory to those from Verweij et al. (2018), who used CD63‐pHluorin to demonstrate that histamine‐induced EV release in HeLa cells was Ca^2+^‐independent, suggesting that histamine‐TRPC3 signalling for EV release is likely to be cell‐type specific. Alternatively, Ca^2+^ signalling may specifically induce the release of a subpopulation of CD81+/CD63‐ EVs. Together, our findings contribute to a growing body of work on GPCR and Ca^2+^ signalling in EV biogenesis (Bebelman et al., 2020; Lu et al., 2018). Future studies will help to unravel the EV biogenesis pathway stimulated downstream of Ca^2+^ signalling and H1HR‐TRPC3 activation in OC cells.

In this study, we specifically explored a role for TRPC3 in EV biogenesis in OC cells as increased TRPC3 activity has been shown to promote tumorigenesis in human OC (Yang et al., 2009). The overexpression and/or overactivity of Ca^2+^ channels and transporters is a characterizing feature of cancer (Monteith et al., 2007), yet roles for Ca^2+^‐dependent downstream effectors are poorly understood. Our data show that direct activation of TRPC3 consistently increased OC cell growth, which is in line with previous studies (Tao et al., 2013; Wang et al., 2019; Yang et al., 2009). Additionally, we show that the application of EVs derived from TRPC3‐activated cells also increases OC cell growth. These findings suggest that overactivity of TRPC3 and a subsequent rise is intracellular Ca^2+^ induces the release of EVs capable of increasing OC cell proliferation. It is conceivable that the observed proliferative effect arises through the transfer and incorporation of functional TRPC3 channels into the membrane of recipient cells—as reported for the EV‐mediated transfer of TRPC5 from chemoresistant breast cancer cells (Ma et al., 2014). However, we cannot rule out the possibility that proliferation of recipient cells is caused by the EV‐mediated transfer of the TRPC3 activator. Future experiments aimed to further profile the released EVs and analyse changes in TRPC3 expression and Ca^2+^ signalling in recipient cells will help to shed light on the mechanisms underlying the proliferative effect of TRPC3‐induced EVs.

Our findings are consistent with previous studies that show that disrupted Ca^2+^ signalling induces the release of EVs with enhanced metastatic competence (Lee et al., 2020; Messenger et al., 2018). For example, Ca^2+^ stimulation of a breast carcinoma line induced a ∼10‐fold increase in proteolytic matrix metalloproteinase (MMP) EV release which promoted cell invasion into neighbouring tissues and vasculature (Messenger et al., 2018; Sahai, 2005). Similarly, removal of free extracellular Ca^2+^ stimulated the release of EVs by OC cells that altered recipient cell adhesion and migration (Lee et al., 2020). The EV‐mediated transfer of Ca^2+^ channels can also upregulate Ca^2+^ signalling in recipient cells, and subsequently promote chemoresistance in a chemosensitive cell population as demonstrated for TRPC5 (Ma et al., 2014). Evident from both our data and the existing literature, the interconnected relationship between Ca^2+^ homeostasis, EVs and tumorigenesis poses as a potential node of therapeutic intervention in cancer treatment. Future studies to elucidate the underlying signalling pathway and to further explore a role for TRPC3 in EV‐mediated communication in OC cell growth could provide novel therapeutic targets for the treatment of OC.

## AUTHOR CONTRIBUTIONS

**Elise H. Padbury**: Conceptualization; data curation; formal analysis; investigation; methodology; validation; visualization; writing—original draft preparation. **Štefan Bálint**: Data curation; formal analysis; methodology; writing—review and editing. **Emanuela Carollo**: Data curation. **David R. F. Carter**: Conceptualization; funding acquisition; investigation; methodology; project administration; resources; supervision; writing—review and editing. **Esther B. E. Becker**: Conceptualization; funding acquisition; investigation; methodology; project administration; supervision; writing—review and editing.

## CONFLICT OF INTEREST STATEMENT

The authors report no conflict of interest.

## Supporting information

Supplementary Information

Supplementary Information

Supplementary Information

Supplementary Information

Supplementary Information
