# Supplementary material for: TRPC3 signalling contributes to the biogenesis of extracellular vesicles
Source: J Extracell Biol. 2023 Dec 25;3(1):e132. doi: 10.1002/jex2.132 (PMC11080740; doi:10.1002/jex2.132)
Supplement: Supplementary file 1 — Supplementary Information [file JEX2-3-e132-s003.pdf]

Supplemental Material

TRPC3 signalling contributes to the biogenesis of extracellular vesicles

Elise H. Padbury[1,2], Štefan Bálint[3], Emanuela Carollo[4], David R. F. Carter[4,5], Esther B. E. Becker[1,2,*]

[1]Nuffield Department of Clinical Neurosciences, University of Oxford, Oxford, UK

[2]Kavli Institute for Nanoscience Discovery, University of Oxford, Oxford, UK

[3]Kennedy Institute of Rheumatology, University of Oxford, Oxford, UK

[4]Department of Biological and Medical Sciences, Oxford Brookes University, Oxford, UK

[5]Current address: Evox Therapeutics Limited, Oxford, UK

*Correspondence to esther.becker@ndcn.ox.ac.uk

Supplemental Methods

Immunoblotting for EV markers

For EV protein extraction, 20-50µl of concentrated EV samples were lysed with an equal volume of 2x RIPA buffer (Sigma-Aldrich®) in PBS. EV lysates were incubated on ice for 10 min prior to centrifugation at 14,000 *g* for 20 min at 4°C. Cellular protein extraction detailed in Methods and Materials. Western blots were run using the same protocol as detailed in Methods and Materials. For cell and EV samples, an equal concentration of 3-50ug total protein was loaded. As minimal protein was detected in the serum-free media control, an equal volume to the EV sample was used. DTT was added to samples at a concentration of 0.4mM when blotting for Cytochrome C and GM130. Antibodies used were mouse anti-TSG101 (1:500, Abcam, ab83), mouse anti-CD81 (1:1,000, Abcam, ab79559), mouse anti-ALIX (1:2,500, Abcam, ab117600), rabbit anti-CD9 (1:1,000, Cambridge Biosciences, EXOAB-CD9A-1), rabbit anti-cytochrome C (1:1,000, Abcam, ab79559), rabbit anti-GM130 (1:1,000, Abcam, ab52649) and HRP-conjugated anti-mouse (1:20,000, Promega, W4021) or anti-rabbit (1:5,000, Promega, W4011) secondary antibodies.

Immunogold labelling

For immunolabelling of CD81 on SKOV3-EVs, 8µl of EV concentrate was deposited on glow discharged, carbon-coated nickel grids (Agar Scientific) for 2 min before grids were blocked in 1% BSA in PBS for 1hr at RT. Grids were then incubated with mouse anti-CD81 (1:100, Abcam, ab79559) in 1% BSA in PBS for 1h at RT. Grids were next washed three times for five minutes per wash in 1% BSA in PBS, before being incubated for 1h at RT with 10nm gold-conjugated anti-mouse (1:50, Abcam, ab27241) in 1% BSA in PBS. After washing steps were repeated, samples were fixed in 2.5% glutaraldehyde (EM grade) in 0.1M phosphate buffer. Finally, grids were washed twice in ddH_2O and negatively stained with 2% uranyl acetate for 10 sec. Grids were left to airdry before imaging. Immunogold-labelled EVs were imaged on a Jeol JEM-1400 Flash Transmission Electron Microscope, using a Gatan OneView 16 Megapixel camera at 100kV.

Immunocytochemistry

SKOV3 cells were seeded on sterilised ø 12-mm coverslips and incubated overnight. The following day, cells were washed in PBS and PM staining was performed using Membrite Fix 680/700 (Biotium) according to the manufacturers protocol. Cells were fixed in 4% PFA (20 min), permeabilised with 0.4% Triton X-100 (20 min) and blocked with TBST (0.2%) containing 10% skim milk powder and 1% normal goat serum (1 h). Coverslips were incubated overnight at 4°C with mouse anti-CD81 (1:100, Thermo Fisher Scientific, 10630D) and rabbit anti-RAB5

(1:200, Cell Signalling Technology, C8B1), or rabbit anti-RAB7 (1:100, Cell Signalling Technology, D95F2). Anti-mouse Alexa Fluor 488 (CD81) (Thermo Fisher Scientific, A1101) and anti-rabbit Alexa Fluor 594 (RAB5/RAB7) (Thermo Fisher Scientific, A11037) secondary antibodies were added the following day and incubated for 2 h at RT, before coverslips were mounted onto glass slides with Vectashield Antifade Mounting Medium with DAPI (Vector Laboratories). Coverslips were imaged on a Zeiss LSM980 Airyscan 2 upright microscope with the Zeiss 20x 0.8 NA Dry objective. The JACOPx Plugin on ImageJ FIJI[1] was used to calculate Pearson's correlation coefficients.

For H1HR immunostaining, SKOV3, OVCAR3 and OVCAR5 cells were seeded, fixed, permeabilised and blocked as above, without Membrite staining. Cells were incubated overnight at 4°C with mouse anti-HRH1 primary antibody (1:50, Santa Cruz Biotechnology, sc-374621). Anti-mouse Alexa Fluor® 594 (Thermo Fisher Scientific, A-11005) secondary antibody was added to cells the following day and incubated for 30 min at RT, before coverslips were mounted onto glass slides using Prolong Gold Antifade DAPI mounting media (Thermo Fisher Scientific). Cells were imaged using a Zeiss Axio Imager 72 upright microscope fitted with an ORCA-Flash 4.0 Digital CMOS camera (Hamamatsu).

qPCR

Total RNA was extracted from SKOV3, OVCAR3 and OVCAR5 cell lines using Direct-zol RNA MiniPrep Kit (ZymoResearch) following the manufacturer's instructions. A total of 300ng RNA was DNAse I treated prior to reverse transcription, which was performed using the HighCapacity cDNA Reverse Transcription Kit (Thermo Fisher Scientific). PCR reactions were performed with iTaq Universal SYBR Green Supermix (Biorad) using the CFX96 TouchTM thermal cycler (Bio-Rad) with the following thermal profile: 30s hot start at 95°C, 35 cycles of 5s at 95°C and 30s at 60°C. Primers for H1HR and β-Actin (ACTB) are shown in Table S1. The fold difference in expression between H1HR and ACTB was calculated using the ΔΔCq method.

Table S1. Primers used for qPCR analysis of H1HR and β-Actin.

**Primer**	**Sequence 5' to 3'**
H1HR forward	TGGTGGTGGATCTGTCTTGA
H1HR reverse	CCGGTTGACGGCTACATAGT
ACTB forward	GGCACCCAGCACAATGAAG
ACTB reverse	CATACTCCTGCTTGCTGATCCA

Supplemental Figures

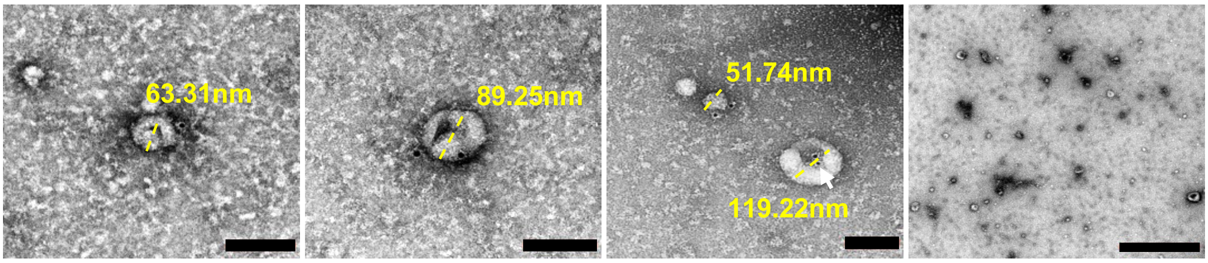

Figure S1. CD81 is enriched on the surface of SKOV3-EVs.
Representative immunogold (anti-CD81) negative stained electron micrographs of SKOV3 EVs. EV diameter measured and shown in yellow. Arrows indicate 10nm gold particles. Scale bars 100nm and 1000nm (far right image only).

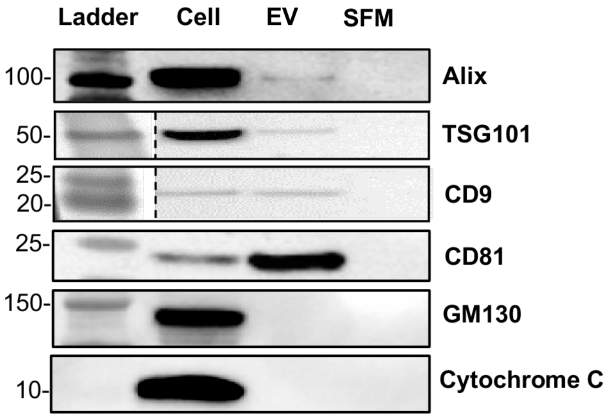

Figure S2. Western blot characterisation of SKOV3-EVs.
Western blotting of EV protein markers (TSG101, Alix, CD9, CD81) in SKOV3 cell lysate and purified EVs. EV lysates were clear of negative markers GM130 and Cytochrome C. SFM: nonconditioned media control.

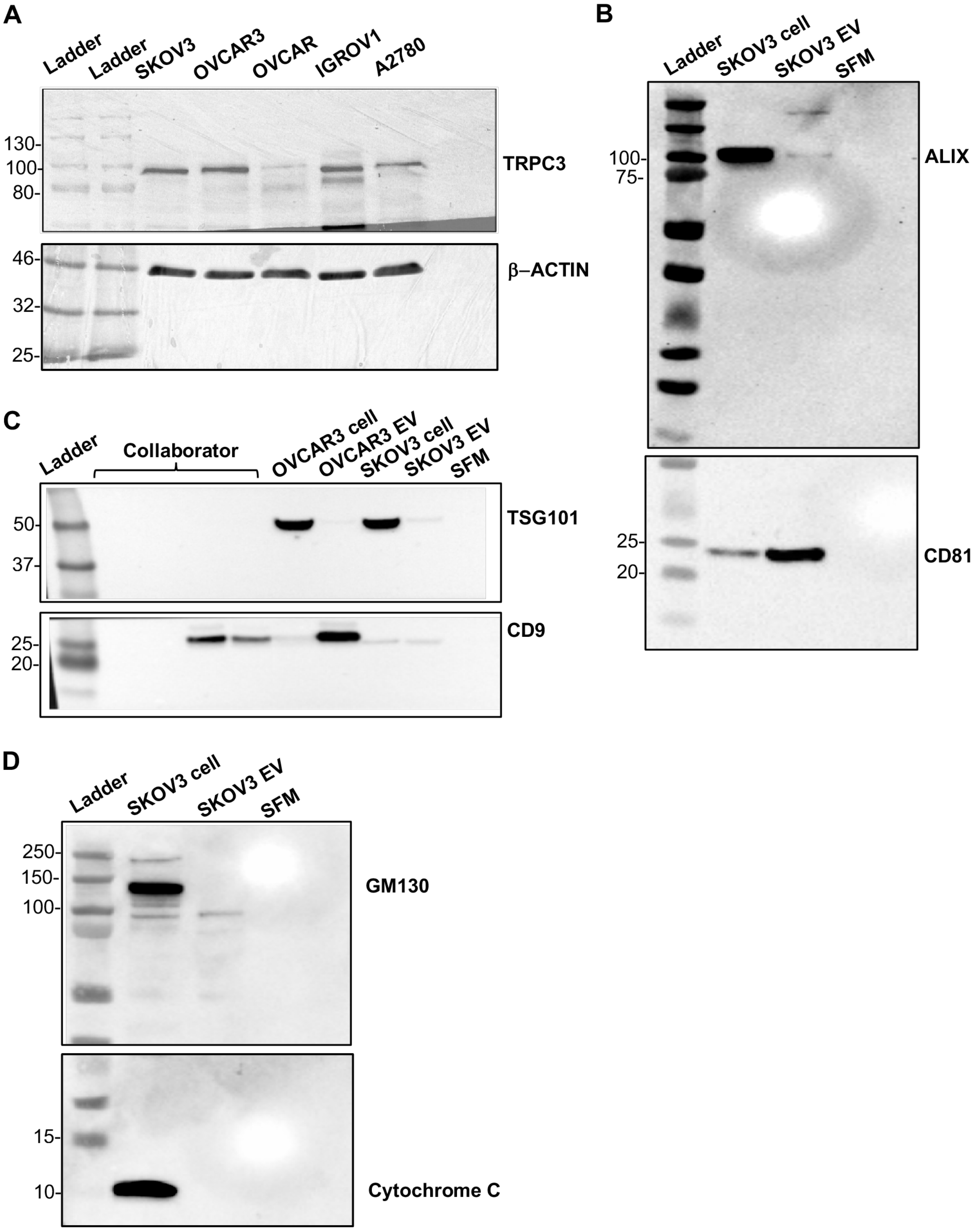

Figure S3. Full blots for all Western blots.

(A) TRPC3 and b-actin expression in ovarian cancer cell lines: SKOV3, OVCAR3, OVCAR5, IGROV1, A2780. **(B)** The expression of EV markers ALIX and CD81 in SKOV3 cell, EV and serum-free media control (SFM). **(C)** The expression of EV markers TSG101 and CD9 in SKOV3 cell, EV and serum-free media control (SFM). Unrelated collaborator samples, OVCAR3 cell and EV samples also shown on full blot. **(D)** The expression of the mitochondrial marker cytochrome C and Golgi marker GM130 in SKOV3 cell, EV and serum-free media control (SFM).

A

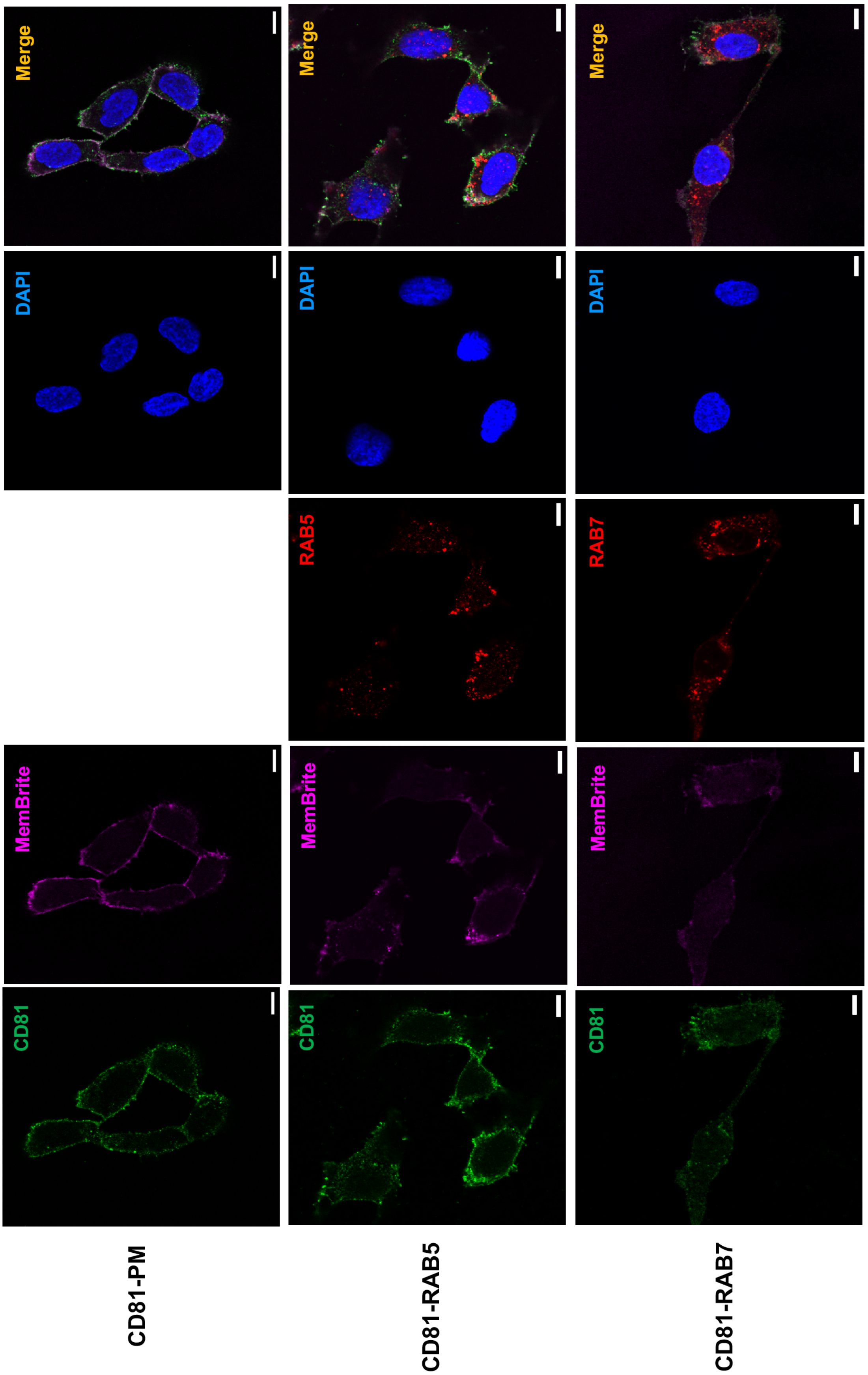
CD81-PM
CD81-RAB5
CD81-RAB7
CD81
MemBrite
RAB5
RAB7
DAPI
Merge

B

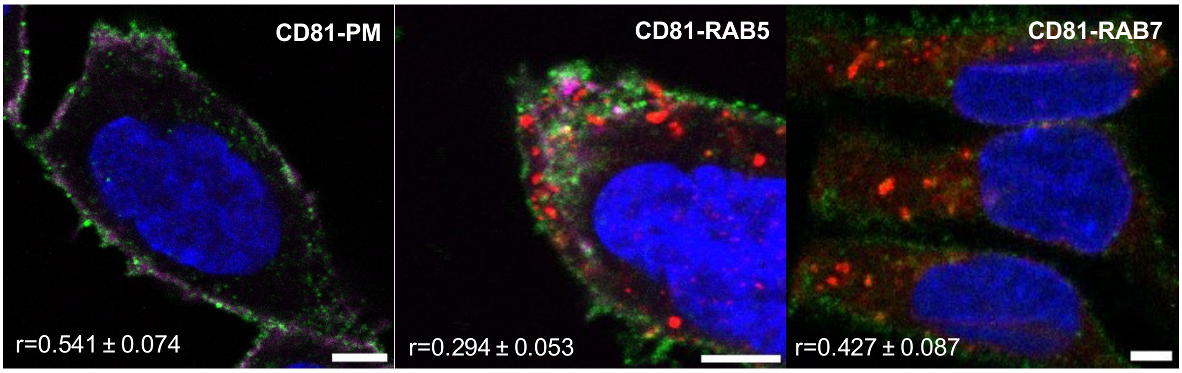

Figure S4. Immunocytochemistry of CD81 expression in SKOV3 cells.
(A) Representative single channel and compositive immunofluorescence images of CD81 expression (green) with Membrite PM stain (magenta), Rab5/RAB7 endosomal markers (red) and DAPI (blue). Scale bars 10μm. The missing panel is intentional as no RAB5/RAB7 staining was used in the top row. **(B)** Close-up compositive images of CD81 (green) colocalisation with Membrite PM stain (magenta) (left), RAB5 (red) (middle), and RAB7 (red) (right). DAPI staining in blue. R values represent mean±SD of Pearson's correlation coefficient of ≥20 cells from two independent experiments. Scale bars 5μm.

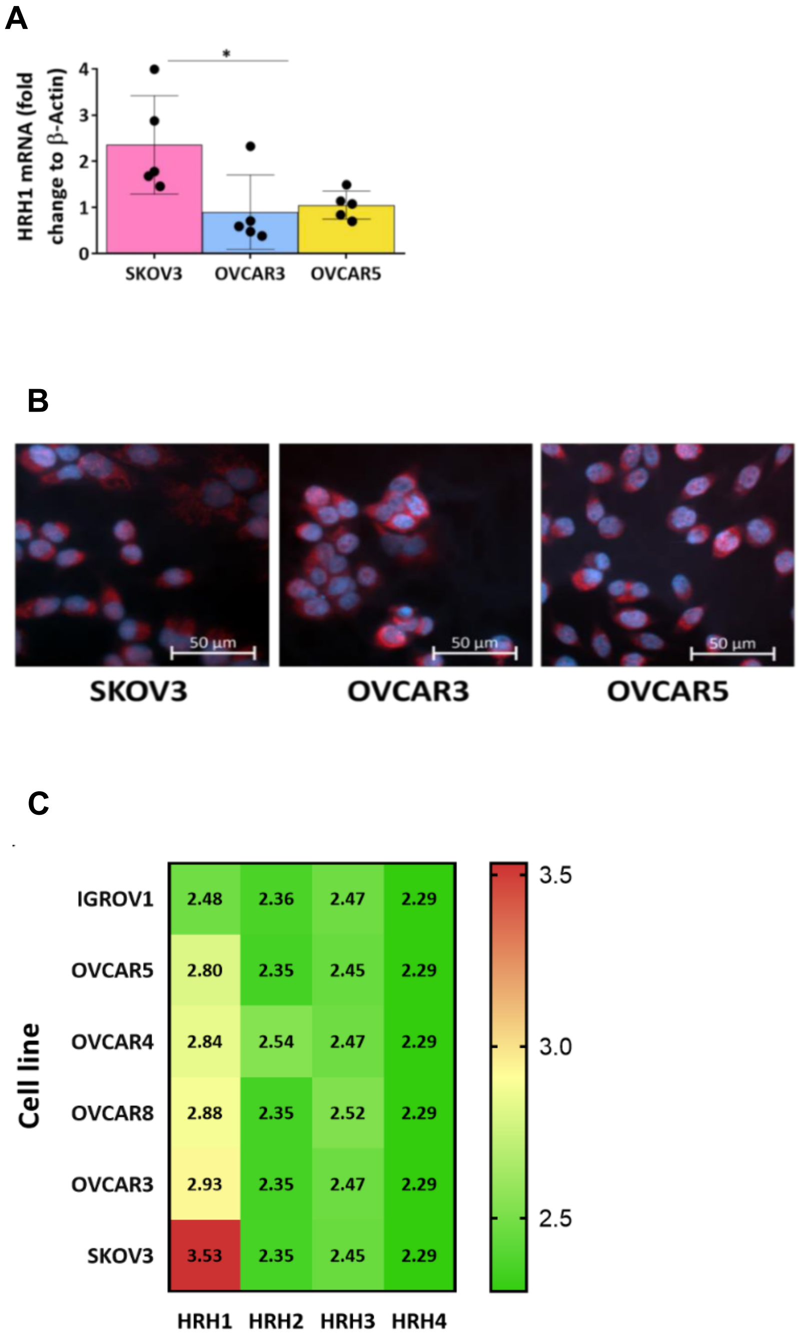

Figure S5. H1HR expression in SKOV3 cells compared to multiple OC cell lines.
(A) RT-qPCR analysis showing the relative quantity of H1HR (normalized on β-Actin) in SKOV3, OVCAR3 and OVCAR5 cell lines. Data represents mean±SD of five independent experiments with statistical significance tested by one-way ANOVA and Tukey's multiple comparisons test. **p*<0.05. **(B)** Representative immunofluorescence images of H1HR (red) and DAPI (blue) in SKOV3, OVCAR3 and OVCAR5 cell lines. Scale bars 50µm. **(C)** Heat-map created from the Cell Miner (GSE32474) dataset showing the mRNA expression levels of H1HR, H2HR, H3HR and H4HR in six OC cell lines.

A

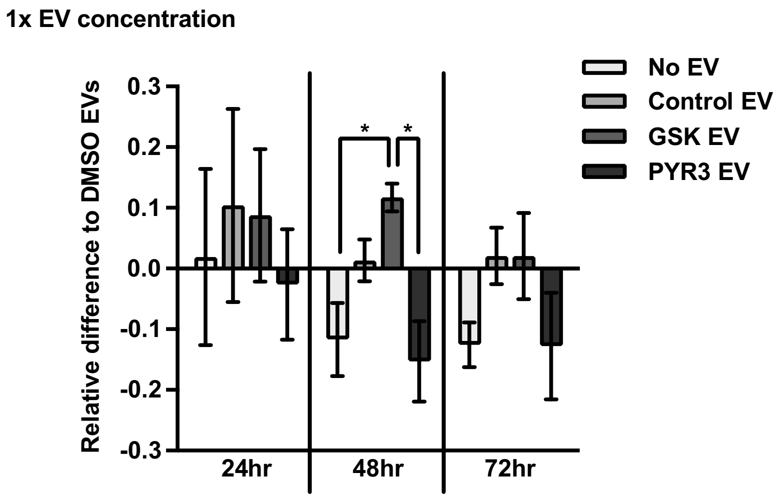

B

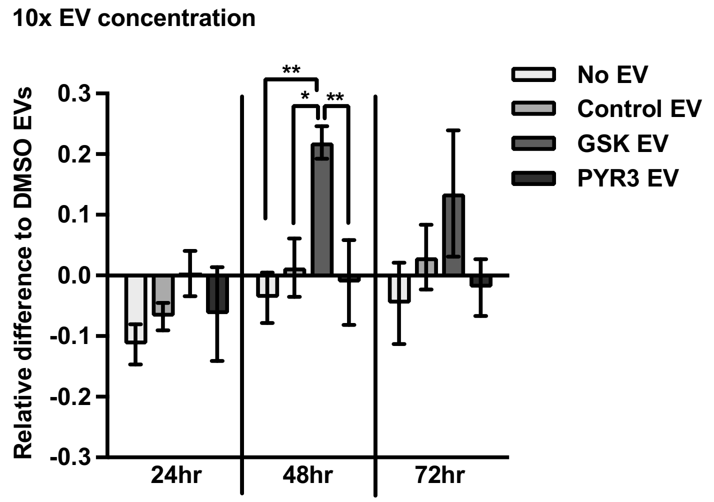

Figure S6. TRPC3 activator-induced EVs increase growth of recipient SKOV3 cells.
(A,B) EVs were extracted from SKOV3 cells treated with PBS (Control EV), 0.3μM GSK1702934A (GSK EV), 3μM PYR3 (PYR3 EV) or DMSO. EVs or PBS (No EV) were applied to naïve SKOV3 cells and recipient cells were counted 24, 48 and 72 hr post-EV treatment. Data shows the relative difference in total cell number between all treatment groups and DMSO EVs (0) at all time-points. (A) 1x and (B) 10x EV treatment concentrations (mean±SEM of three independent experiments, each consisting of three technical replicates. Statistical significance tested by one-way ANOVA and Tukey's multiple comparisons test. *p≤0.05, **p≤0.01).

Supplemental Videos

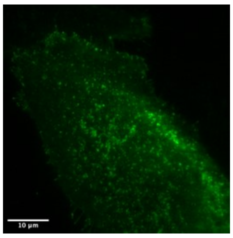

Video S1. TIRF imaging of CD81+ events in untreated SKOV3 cells. SKOV3 cells transfected with the CD81-pHluorin plasmid were imaged at 0.4 fps over a 5-minute time course.

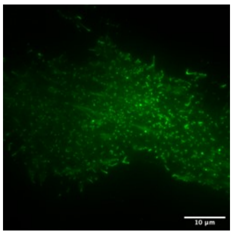

Video S2. TIRF imaging of CD81+ events in ionomycin-treated SKOV3 cells. SKOV3 cells transfected with the CD81-pHluorin plasmid were stimulated with 1.25μM ionomycin and imaged at 0.4 fps over a 5-minute time course immediately following stimulation.

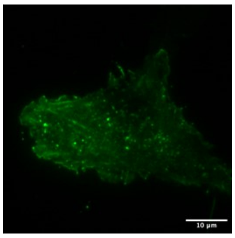

Video S3. TIRF imaging of CD81+ events in histamine-treated SKOV3 cells. SKOV3 cells transfected with the CD81-pHluorin plasmid were stimulated with 100μM histamine and imaged at 0.4 fps over a 5-minute time course immediately following stimulation.

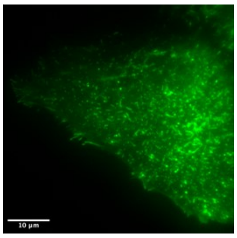

Video S4. TIRF imaging of CD81+ events in SKOV3 cells treated with 0.1μM GSK1702934A. SKOV3 cells transfected with the CD81-pHluorin plasmid were stimulated with 0.1μM TRPC3 activator GSK1702934A and imaged at 0.4 fps over a 5-minute time course immediately following stimulation.

Supplemental References

1. Schindelin, J, Arganda-Carreras, I, Frise, E, et al. Fiji: an open-source platform for biological-image analysis. Nature Methods 2012;9(7):676-682.
